# Decrease expression of microRNA-744 promotes cell proliferation by targeting c-Myc in human hepatocellular carcinoma

**DOI:** 10.1186/1475-2867-14-58

**Published:** 2014-06-22

**Authors:** Feng Lin, Ruliang Ding, Shuang Zheng, Dongyi Xing, Weiwen Hong, Zhijun Zhou, Jie Shen

**Affiliations:** 1 Department of General Surgery, Taizhou First People's Hospital, Taizhou, Zhejiang Province 318020, P.R. China; 2 Department of General Surgery, The Affiliated Huangyan Hospital of Wenzhou Medical University, Taizhou, Zhejiang Province 318020, P.R. China

**Keywords:** Hepatocellular carcinoma (HCC), miR-744, c-Myc, Cyclin D1, Tumor suppressors

## Abstract

**Background:**

MicroRNAs (miRNAs) are a large group of post-transcriptional gene regulators that potentially play a critical role in tumorigenesis. Increasing evidences indicate that miR-744 deregulated in numerous human cancers including hepatocellular carcinoma (HCC). However, its role in HCC carcinogenesis remains poorly defined. In this study, we investigated the roles of miR-744 in tumor growth of HCC.

**Methods:**

Quantitative reverse-transcription polymerase chain reaction (qRT-PCR) was conducted to detect the expression of miR-744 and Immunohistochemistry was performed to detect expression of c-Myc in HCC specimens and adjacent normal tissues. The biological functions of miR-744 were determined by cell proliferation and cell cycle assay. Furthermore, cell lines transfected with miR-744 mimics were analyzed *in vitro*. Luciferase reporter assays was performed to confirm whether miR-744 regulated the expression of c-Myc.

**Results:**

Our results showed that the expression of miR-744 was frequently down-regulated in both HCC tissues and cells. Furthermore, restoration of miR-744 in HCC cells was statistically correlated with decrease of cell growth and restored G1 accumulation. Luciferase assay and Western blot analysis revealed that c-Myc is a direct target of miR-744. Down-regulation of miR-744 and up-regulation of c-Myc were detected in HCC specimens compared with adjacent normal tissues. Moreover, restoration of miR-744 rescues c-Myc induced HCC proliferation.

**Conclusions:**

Our data suggest that miR-744 exerts its tumor suppressor function by targeting c-Myc, leading to the inhibition of HCC cell growth. miR-744 may serve as a potentially useful target for the miRNA-based therapies of HCC in the future.

## Background

miRNAs are one family of small (~22 nucleotides), non-coding RNAs which play an important role in regulating diverse biological processes, such as proliferation, differentiation, apoptosis, development and metabolism [1]. Increasing evidences indicate that some miRNAs act as either tumor suppressors or oncogenes in the tumorigenesis of cancer and have been potential biomarkers for tumor therapy, diagnosis, prognosis [2-5].

HCC is one of the most common cancers in the world which accounts for ~700 000 deaths annually and with an estimated 21 000 new diagnosed cases [6]. Much efforts have been spent on the study of the biological mechanism of HCC cells to develop effective treatment strategies. Altered miRNA expression in HCC has been found in different studies. Several deregulated miRNAs in HCC such as miR-195 [7], miR-101 [8], miR-122 [9], miR-221 [10], miR-224 [11] miR-223 [12], miR-21 [13] and miR-199 [14] have been shown to regulate cell growth, apoptosis, migration and invasion. These findings suggest that deregulation of miRNA may be associated with tumorigenesis of HCC. More extensive investigations are required to elucidate the role of miRNAs in the development of HCC, and those miRNAs may be employed as novel biomarkers for HCC therapy, diagnosis, prognosis.

miR-744 is significantly deregulated in several cancers, including HCC [14], colon cancer [15], breast cancer [16], and gastric cancer [17]. Because deregulation of miR-744 is common to a number of cancers, it has been hypothesized that miR-744 may play an important role in tumor development and tumorigenesis. However, the function of miR-744 remains unclear, especially in HCC.

In our current study, we evaluated miR-744 expression levels in 40 tumor tissues of patients with HCC and found that miR-744 was significantly down-regulated in HCC. Based on gain-of-function approach, we proved that miR-744 could inhibit HCC cell proliferation in vitro. Furthermore, c-Myc, which is usually overexpressed in a variety of human cancers including HCC, was identified as a direct target of miR-744. Our findings will help to elucidate the functions of miR-744 and their roles in HCC tumorigenesis.

## Results

### miR-744 down-regulated in HCC

**Figure 1:**
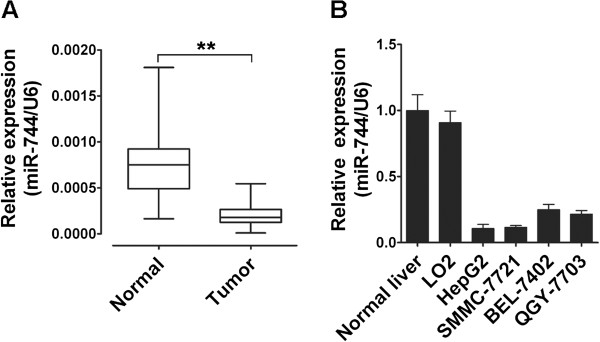
**miR-744 is down-regulated in HCC samples and HCC cell lines. (A)** Expression of miR-744 was measured in 40 HCC samples and normal liver tissues by qRT-PCR, and the expression levels of miR-744 were normalized to U6 RNA expression for subsequent analyses. **(B)** The expression levels of miR-744 were further measured in 5 normal liver tissues, normal liver cell line LO2 and 4 HCC cell lines by qRT-PCR, and the expression levels of miR-744 were normalized to U6 RNA expression for subsequent analyses. The normalized expression of normal liver tissues was set as relative expression 1. The data were subjected to Student's *t*-test. *p < 0.05, **p < 0.01.

To determine the levels of miR-744 in HCC samples and cell lines, total RNAs were extracted from HCC tissues and cell lines, and the expression levels of miR-744 were analyzed using qRT-PCR and normalized against an endogenous control (U6 RNA). As shown in Figure 1A, miR-744 was significantly decreased in HCC tissues versus adjacent normal tissues. It was also shown that miR-744 was down-regulated in 4 HCC cell lines, compared with 5 normal liver tissues and normal liver cell line LO2 (Figure 1B). Taken together, our results revealed that miR-744 was abnormally down-regulated both in human HCC samples and cell lines.

### miR-744 inhibits HCC growth

**Figure 2:**
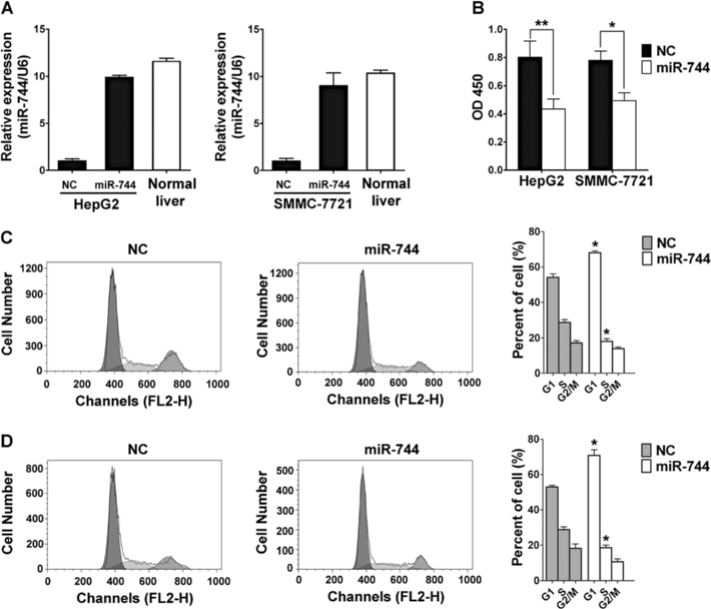
**MiR-744 restoration inhibit the viability of HCC cells. (A)** miR-744 expression was quantified by qRT-PCR analysis. miR-744 mimics restorated miR-744 expression in both HepG2 and SMMC-7721 cells. **(B)** Proliferation assay of HCC cells in response to miR-744 restoration. HepG2 or SMMC-7721 cells were seeded into 96-well plates and incubated in the presence of miR-744 mimics or negative control oligonucleotides. Cell proliferationassay was done after culturing for 72 h. **(C, D)** Cell-cycle distribution was analyzed by FACS analysis. HepG2 **(C)** and SMMC-7721 **(D)** cells were transfected with miR-744 mimics or negative control oligonucleotides. Nocodazole (25 ng/ml) was added 24 h after transfection for another 16 h, then the supernatant was replaced by fresh medium for 6 h. The data were subjected to Student's *t*-test. *p < 0.05, **p < 0.01. NC, negative control oligonucleotides.

We then investigated the biological significance and its underlying mechanisms of the silenced miR-744 in HCC. Cell proliferation is a key character during tumorigenesis. However, the association of miR-744 with HCC cell proliferation is unknown. miR-744 mimics or negative control oligonucleotides was transiently transfected into human HCC cell lines that have lowly endogenous expression levels of miR-744 (Figure 1B). Expression of miR-744 was verified by qRT-PCR. qRT-PCR results determined that transfection of miR-744 restored its expression in HepG2 and SMMC-7721 cells (Figure 2A). In cell proliferation assay, restoration of miR-744 in HepG2 and SMMC-7721 cells resulted in significant suppression of cell proliferation, the proliferation rate was suppressed in HepG2 and SMMC-7721 cells after transfection with miR-744, and the inhibitory efficiencies were 45.8% and 36.7%, respectively (Figure 2B).

Cell cycle analysis was performed to determine whether the effect of miR-744 on cell proliferation was due to cell cycle arrest. The result showed that the cell cycle was arrested in G1 phase, with 69.17% ± 8.74% of miR-744-transfected cells in G0/G1 versus 54.02% ± 7.16% of negative control oligonucleotides-transfected cells in HepG2 cells (Figure 2C). Similar effects of miR-744 were found in SMMC-7721 cells (Figure 2D), with 70.95% ± 8.92% of miR-744-transfected cells in G0/G1 versus 53.32% ± 8.52% of negative control oligonucleotides-transfected cells. These results demonstrate that miR-744 regulates proliferation of HCC cells.

### miR-744 targets c-Myc

**Figure 3:**
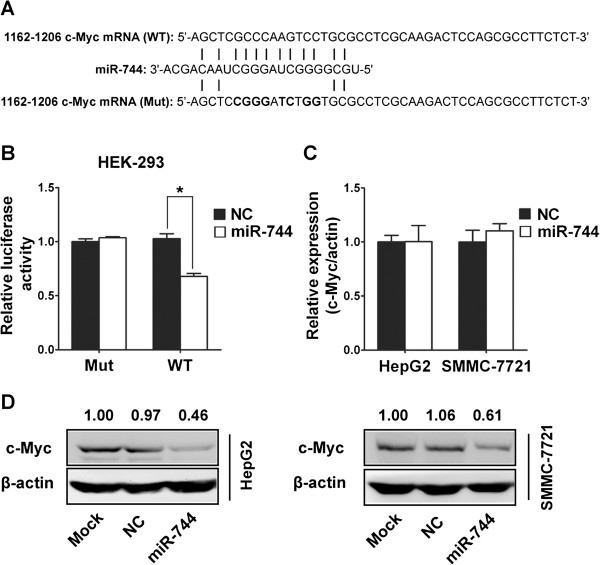
**miR-744 directly targets and regulates c-Myc expression in HCC cells. (A)** Wild-type (WT) and mutant (Mut) of putative miR-744 targeting sequences in c-Myc mRNA. Mutant sequences were shown in bold type. **(B)** Analysis of luciferase activity. HEK-293 cells were cotransfected with miR-744 mimics or control oligo nucleotides, pRL-TK and firefly luciferase reporter plasmid containing putative miR-744 targeting sequences of c-Myc. pRL-TK was cotransfected as an internal control to correct the differences in both transfection and harvest efficiencies. The firefly luciferase activity of each sample was normalized to the Renilla luciferase activity. The normalized luciferase activity control oligo nucleotides was set as relative luciferase activity 1 respectively. **(C)** Effects of miR-744 mimics on the endogenous c-Myc mRNA levels. HepG2 and SMMC-7721 cells were cotransfected with miR-744 mimics or negative control oligonucleotides. Forty-eight hours after transfection, cells were isolated, the expression of c-Myc was analyzed by qRT-PCR. **(D)** Effects of miR-744 on the endogenous c-Myc protein levels. Forty-eight hours after transfection with the miR-744 mimics or negative control oligonucleotides, HepG2 and SMMC-7721 cells were analyzed by Western blotting, with β-actin as an internal control. The value up each lane indicates the c-Myc expression in oligonucleotides-transfected cells compared with mock-transfected (with only transfection reagent) cells. The data were subjected to Student's *t*-test. *p < 0.05, **p < 0.01. NC, negative control oligonucleotides.

As miRNAs function mainly through inhibition of target genes, the target gene of miR-744 that functions in HCC pathogenesis was further analyzed. We checked the newly published CLASH data [18], we found that about 402 genes were targeted by miR-744 in HEK293 cells. Among these genes, we focus on c-Myc gene, a central component of the MAPK/ERK pathway, which is involved in many cellular processes including cell growth, cell differentiation, apoptosis and other cellular functions [19-21]. c-Myc is a very strong proto-oncogene and it is often found to be up-regulated in many types of cancers including HCC [22,23]. From the CLASH data in KEK293 cells, the potential targeting sequence for miR-744 with a calculated energy of −24.1 kcal/mol is within the protein coding region of c-Myc mRNA from 1162 to 1206. This is clearly distinct from the recently reported c-Myc targeting miR-24 [24], miR-145 [25] and let-7a [26], but is similar to miR-185-3p [27]. To confirm whether miR-744 could regulate the expression of c-Myc, we first performed luciferase reporter assays in HEK293 cells. We created luciferase reporter plasmid with wild type or mutant targeting sequence of c-Myc mRNA (Figure 3A), which were cotransfected with miR-744 mimics or negative control oligonucleotides into HEK293 cells for 48 h, followed by measurement of luciferase activity in transfected cells. Our results showed that the reporter plasmid with wild type targeting sequence of c-Myc mRNA caused a significant decrease in luciferase activity in cells transfected with miR-744, whereas reporter plasmid with mutant sequence of c-Myc produced no change in luciferase activity (Figure 3B). Then, we explored whether the endogenous c-Myc in HCC cells was regulated similarly. HepG2 and SMMC-7721 cells were transfected with miR-744 mimics or negative control oligonucleotides, and c-Myc mRNA and protein levels were examined by qRT-PCR and western blotting, respectively. c-Myc mRNA expression was not affected by miR-744 (Figure 3C). The level of c-Myc protein was consistently and substantially down-regulated by miR-744 (Figure 3D).

**Figure 4:**
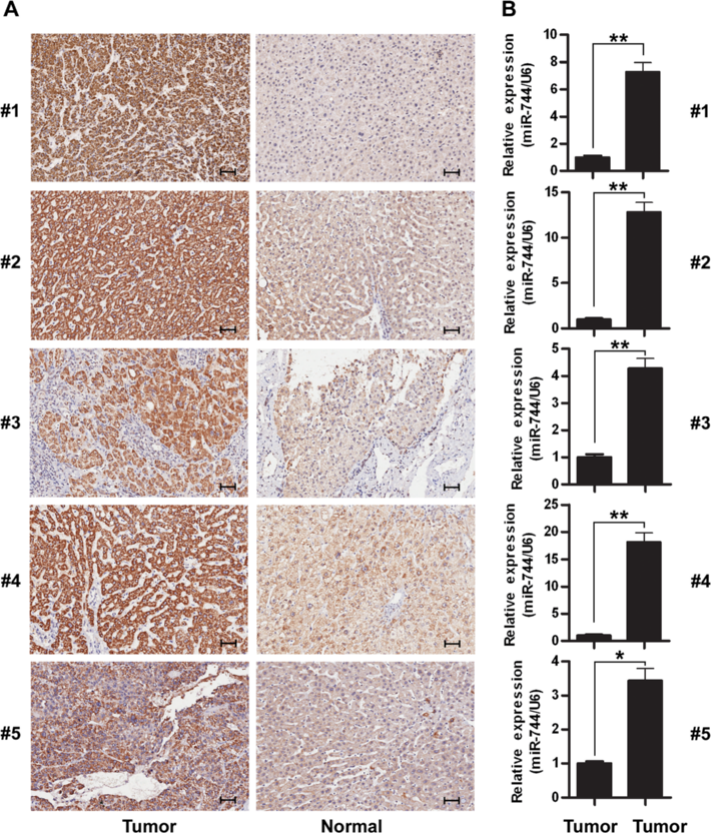
**Expression analyses of miR-744 and c-Myc in HCC tissues. (A)** Analysis of c-Myc expression in five HCC tissues and adjacent normal tissues by IHC. Brown signal in IHC was considered as positive staining for c-Myc. Scale bar = 50 μm. **(B)** Analysis of miR-744 expression in the same HCC tissues and adjacent normal tissues by qRT-PCR. The data were subjected to Student's *t*-test. *p < 0.05, **p < 0.01.

Consistently, HCC tissues with low miR-744 showed much higher c-Myc expression, compared with normal liver tissues with high level of miR-744 but lower c-Myc expression (Figure 4A,B). Taken together, our results demonstrated that c-Myc was a direct target of miR-744 in HCC cells.

### Restoration of miR-744 rescues c-Myc induced HCC proliferation

**Figure 5:**
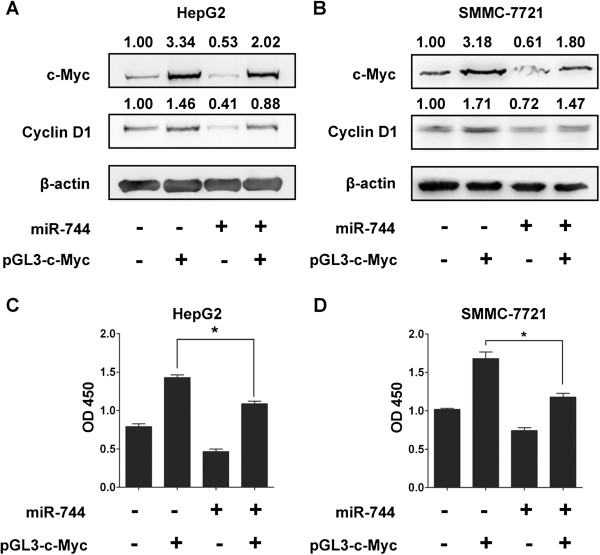
**miR-744 inhibits c-Myc-dependent activity and cell proliferation in HCC cells. (A, B)** miR-744 inhibits c-Myc-dependent transcription of the c-Myc target gene Cyclin D1. HepG2 and SMMC-7721 cells were transfected with miR-744 and/or pGL3-c-Myc, and the expression of c-Myc and Cyclin D1 were analyzed by Western blotting. The value up each lane indicates the protein expression with β-actin as an internal control. **(C, D)** miR-744 inhibits c-Myc-dependent cell proliferation. HepG2 and SMMC-7721 cells were transfected with miR-744 and/or pGL3-c-Myc, and cells proliferation was measured by CCK-8 analysis. The data were subjected to Student's *t*-test. *p < 0.05.

c-Myc is a nuclear transcriptional factor and most of its targeting genes including Cyclin D1 are crucial in the regulation of cell growth, differentiation, apoptosis and other cellular functions [28]. We had resulted that restoration of miR-744 could suppress the growth and proliferation of HCC cells and c-Myc was a direct target of miR-744. So we hypothesized that miR-744 regulated cell growth in HCC cells by targeting c-Myc. One endogenous targets Cyclin D1 of the c-Myc in cancer cells were tested. As expected, ecoptic c-Myc in HepG2 cells enhanced the accumulation of Cyclin D1, while restoration the miR-744 expression in HepG2 cells partially inhibited expression of Cyclin D1 (Figure 5A). In addition, miR-744 also inhibited the c-Myc-dependent expression of endogenous Cyclin D1 in SMMC-7721 cells (Figure 5B). In line with these results, miR-744 partially alleviated c-Myc-induced cell proliferation in HepG2 and SMMC-7721 cells (Figure 5C and D). Taken together, these results demonstrated that miR-744 could regulate HCC cells growth partially through targeting c-Myc.

## Discussion

In this study, we focused on miR-744 which was decreased in HCC tissues and four HCC cell lines. Our results agreed with the previously study in HCC [14]. Additional experiments demonstrated that restoration of miR-744 significantly decreased the proliferation of the HepG2 and SMMC-7721 cell lines. The decreased proliferation of the HCC cell lines resulted from inhibiting cell cycle progression in vitro. Moreover, we identified c-Myc, which is an oncogenic transcription factor, as a direct and functional target of miR-744 in HCC cells.

It has been well established that miRNAs play critical roles in the development, differentiation, proliferation, apoptosis, invasion and metastasis of a variety of cancers. Recently, deregulation of miRNA in cancer cells and their roles in tumorigenesis have been increasingly investigated [2,29,30]. miR-744 has been reported to be frequently deregulated in various kinds of cancers. miR-744 was able to inhibit the proliferation of breast cancer cells by targeting eukaryotic translation elongation factor 1A2 (eEF1A2) [16]. Previous studies identified a panel of serum miRNAs including miR-744 as potential biomarkers for gastric cancer detection [17]. Long-term overexpression of miR-744 may cause chromosomal instability and inhibit tumor growth by prolonging activation of Ccnb1 in mouse [31]. However, the role of miR-744 in cancers especially in HCC is not very much known. In the present study, our data show that compared with adjacent normal liver tissues, miR-744 expression in HCC is significantly down-regulated, suggesting that miR-744 is a candidate tumor suppressor in the pathogenesis of HCC.

c-Myc is a nuclear transcriptional factor and most of its targeting genes are crucial for ribosome biogenesis and protein synthesis including Cyclin D1 [32-34], which are indispensable for cell growth, proliferation, and development. For its biological importance, c-Myc is carefully regulated at transcriptional, post-transcriptional, translational and posttranslational levels [19]. However, the level of c-Myc are highly expressed in most human cancers including HCCs. Although a variety of protein-associated mechanisms have been shown to be involved for its highly expression in cancers [19], several miRNA such as miR-24, miR-22, miR-145, let-7a,miR-34a, miR-185-3p were reported as regulators of c-Myc [24]. These miRNAs can inactivate c-Myc by targeting its mRNA with different mechanisms. miR-24, miR-22, miR-145, let-7a and miR-34a bind to distinct seeding sequences residing in the 3′-UTR of c-Myc mRNA. However, miR-185-3p and miR-744 in our study have been reported to target protein-coding sequence of c-Myc mRNA.

Our findings demonstrated that miR-744 was frequently down-regulated in HCC cell lines and clinical samples. Our data demonstrated for the first time that restoration of miR-744 significantly inhibited HCC cells proliferation through down-regulating c-Myc protein level. Moreover, miR-744 down-regulated c-Myc protein level through targeting its protein-coding sequence but not 3′UTR. Our identification of c-Myc as a target of miR-744 provides new insights into the mechanisms underlying HCC proliferation and miR-744 have potential as novel therapeutic targets for the treatment of HCC.

## Conclusions

In summary, our data show that miR-744 is down-regulated in HCC tissues compared with normal liver tissues. miR-744 directly downregulates c-Myc and inhibits HCC cell proliferation. These data indicate that miR-744 may serve as a new target for cancer therapy.

## Materials and methods

### Tissue samples and cell lines

Forty primary HCC tissues and adjacent normal liver tissues were obtained from the Department of General Surgery, Taizhou First People's Hospital (Zhejiang, China) for qRT-PCR analysis. The histopathological diagnosis of HCC was confirmed by the hospital’s Pathology Department according to the criteria of the World Health Organization after the operation. All tissue samples were from the surgical removal and immediately snap frozen in liquid nitrogen and stored in liquid nitrogen until use. The study protocol was approved by Ethical Committee of Biomedicine Research from General Hospital of Taizhou Military Command. Informed consent was obtained from all patients. All patients had no history of radiotherapy or chemotherapy preoperatively. All clinicopathologic and biological data were available for those patients. Patients’ characteristics of clinical-pathologic features were listed in Table 1. Additionally, five normal liver tissues were obtained from adjacent liver tissues of contusion and laceration in traumatic liver injury patients. All HCC cell lines (HepG2, SMMC-7721, QGY-7703, BEL-7402) and human hepatic cell lines LO2 used in this study were purchased from the cell bank of the Chinese Academy of Sciences and grown were cultured in DMEM media containing 10% FBS. HEK293 cells were purchased from ATCC and grown were cultured in DMEM media containing 10% FBS. Cells were maintained at 37°C in a humidified atmosphere with 5% CO_2_.

**Table 1:** Patients’ characteristics of clinical-pathologic features

**Characteristics**	**No. of patients (n = 40)**	**Percent (%)**
Age at diagnosis (year)		
≤ 40	8	20.0
> 40	32	80.0
Sex		
Male	35	87.5
Female	5	12.5
Tumor size (cm)		
≤ 2	5	12.5
> 2 and ≤ 5	24	60.0
> 5	11	27.5

### RNA isolation and qRT-PCR

Total RNA was isolated from HCC tissues, adjacent normal liver tissues, HCC cell lines and human hepatic cell lines LO2 using Trizol according to the manufacturer’s instructions. qRT-PCR-based detection of mature miR-744 was performed as described previously [14]. U6 small RNA was used as an internal control for normalization and quantification of miR-744 expression. All experiments were done in triplicate. All primers were listed in Table 2.

**Table 2:** All primers used in this study

**Name**	**Primer Sequence**
U6 F	5′ - CTCGCTTCGGCAGCACA -3′
U6 R	5′- AACGCTTCACGAATTTGCGT -3′
c-Myc (WT) F	5′-AAACTAGTGAGCTCGCCCAAGTCCTG -3′
c-Myc (WT) R	5′-GGAAGCTTAGAGAAGGCGCTGGAG -3′
c-Myc (MUT) F	5′-AAACTAGTGAGCTCCGGGATCTGGTG -3′
c-Myc (MUT) R	5′-GGAAGCTTAGAGAAGGCGCTGGAG -3′
c-Myc (pGL3) F	5′-AAAGGTACCATGCCCCTCAACGTTAGCTTCACC-3′
c-Myc (pGL3) R	5′-AAACTCGAGTTACGCACAAGAGTTCCGTAGC-3′
miR-744 RT	5′-GTCGTATCCAGTGCAGGGTCCGAGGTATTCGCACTGGATACGATGCTGT-3′
miR-744 F	5′- AATGCGGGGCTAGGGCTA-3′
miRNA Universal R	5′- GTGCAGGGTCCGAGGT-3′

Abbreviations: RT, reverse-transcription primer; F, forward primer; R, reverse primer; WT, wild type; MUT, mutant.

### Oligonucleotides and plasmids transfection

RNA oligos were chemically synthesized and purified by Genepharma Co. Ltd., (Shanghai, China). Sense sequence of human miR-744 mimics was 5′- UGC GGG GCU AGG GCU AAC AGC A -3′ and antisense sequence was 5′- UGC UGU UAG CCC UAG CCC CGC A-3′. Negative control oligonucleotides was 5′-AAU UCU CCG AAC GUG UCA CTT-3′ and 5′-GUG ACA CGU UCG GAG AAU UTT-3′. The transfections were performed with INTERFERin reagent (Polyplus-transfection). The final concentration of miRNA was 50 nM.

To generate pGL3-c-Myc constructs, the sequence of c-Myc mRNA was amplified by the primers listed in Table 2. The fragments were inserted into pGL3 with the designed cutting sites: *Kpn*I and *Xho*I. The transfections were performed with Lipofectamine 2000 reagent (Invitrogen, USA). The final concentration of plasmids was 100 ng.

### Luciferase reporter assays

Luciferase reporter construct was made by cloning human c-Myc mRNA sequence into pMIR-Report construct (Ambion, Austin, USA). Wild type or mutant c-Myc mRNA fragment (from 1162 to 1206) was amplified and cloned into the luciferase repoter via *Spe*I and *Hind*III sites. All the primers were listed in Table 2. Luciferase reporter assays were performed as previously [14], HEK293 cells plated in a 48-well plate were co-transfected with 50 nM single-stranded miRNA mimics or negative control oligonucleotides, 50 ng of firefly luciferase reporter and 10 ng of pRL-TK (Promega, USA) using the JetPRIME reagent (Polyplus-transfection). Cells were collected 36 hours after last transfection and analyzed using Dual-Luciferase Reporter Assay System (Promega).

### Cell proliferation assay

Cell proliferation was measured using the CCK-8 assay kit (Dojindo Corp., Japan) according to the manufacturer’s protocol. Cells were plated into each well of a 96-well plate and transfected with miRNA mimics or negative control oligonucleotides. On the day of harvest, 10 μl CCK-8 was added to 90 μl of culture medium. The cells were subsequently incubated for 2 hr at 37°C and the optical density was measured at 450 nm. Three independent experiments were performed.

### Western blot

Proteins were separated on a 12% SDS-PAGE gel and transferred onto a nitrocellulose membrane (Bio-Rad, Hercules, USA). The membrane was blocked with 5% non-fat milk and incubated with anti-c-Myc antibody, anti-Cyclin D1 (Santa Cruz, CA) or anti-beta-actin antibody (Sigma, CA, USA). After being washed extensively, a goat anti-mouse secondary antibody (Pierce, IL, USA) was added to the system. The proteins were detected using ECL reagents (Pierce).

### Immunohistochemistry

Paraffin-embedded tissue sections were deparaffinized in xylene and rehydrated in graded series of ethanols followed by heat induced epitope retrieval in citrate buffer (PH 6.0). c-Myc expression were detected using anti-c-Myc polyclonal antibody (Santa Cruz, CA; 1:80). After incubation with a biotinylaed secondary antibody and DAB (Dako, Carpenteria, CA), the slides were rinsed and counterstained with Mayer’ hematoxylin.

### Statistical analysis

All statistical analyses were carried out using the SPSS 16.0 statistical software package. Continuous variables were expressed as mean ± SEM. Differences between groups were calculated with Student’s t test. A two-tailed *P* value test was used with a *P* value of < 0.05 considered statistically significant.

## Abbreviations

HCC: Hepatocellular carcinoma; mRNA: Messenger RNA; qRT-PCR: Quantitative reverse-transcription polymerase chain reaction; miRNA: MicroRNA; IHC: Immunohistochemistry.

## Competing interests

The authors declare that they have no competing interests.

## Authors’ contributions

JS and ZZ conceived and designed the experiments. FL, RD, SZ, DX and WH performed the experiments. JS and FL collected the samples and analyzed the data. FL wrote the paper. All authors are in agreement with the content of the manuscript and this submission. All authors read and approved the final manuscript.
